# Comprehensive Evolutionary Analysis of Complete Epstein–Barr Virus Genomes from Argentina and Other Geographies

**DOI:** 10.3390/v13061172

**Published:** 2021-06-18

**Authors:** Ana Catalina Blazquez, Ariel José Berenstein, Carolina Torres, Agustín Izquierdo, Carol Lezama, Guillermo Moscatelli, Elena Noemí De Matteo, Mario Alejandro Lorenzetti, María Victoria Preciado

**Affiliations:** 1 Laboratorio de Biología Molecular, División Patología, Instituto Multidisciplinario de Investigaciones en Patologías Pediátricas (IMIPP), CONICET-GCBA, Buenos Aires C1425EFD, Argentina; blazquez.a.catalina@gmail.com (A.C.B.); arieljberenstein@gmail.com (A.J.B.); elenadematteo@gmail.com (E.N.D.M.); lorenzetticonicet@gmail.com (M.A.L.); 2 Instituto de Investigaciones en Bacteriología y Virología Molecular (IBaViM), Facultad de Farmacia y Bioquímica, Universidad de Buenos Aires, Buenos Aires C1113AAD, Argentina; caro.torr@gmail.com; 3 Consejo Nacional de Investigaciones Científicas y Técnicas (CONICET), Buenos Aires C1425FQB, Argentina; 4 División de Endocrinología, Centro de Investigaciones Endocrinológicas “Dr. César Bergadá” (CEDIE), CONICET-FEI-GCBA, Buenos Aires C1425EFD, Argentina; aguizquierdo@gmail.com; 5 Unidad de Hepatología, Hospital de Niños Ricardo Gutiérrez, Buenos Aires C1425EFD, Argentina; lezamacarol@hotmail.com; 6 Servicio de Parasitología y Chagas, Instituto Multidisciplinario de Investigaciones en Patologías Pediátricas (IMIPP), CONICET-GCBA, Buenos Aires C1425EFD, Argentina; gfmoscatelli@yahoo.com.ar

**Keywords:** Epstein–Barr virus, next-generation sequencing, evolution rate, geographic variability, EBV Argentina

## Abstract

The sequence variability of the Epstein–Barr virus has been extensively studied throughout previous years in isolates from various geographic regions and consequent variations at both genetic and genomic levels have been described. However, isolates from South America were underrepresented in these studies. Here, we sequenced 15 complete EBV genomes that we analyzed together with publicly available raw NGS data for 199 EBV isolates from other parts of the globe by means of a custom-built bioinformatic pipeline. The phylogenetic relations of the genomes, the geographic structure and variability of the data set, and the evolution rates for the whole genome and each gene were assessed. The present work contributes to overcoming the scarcity of complete EBV genomes from South America and is the most comprehensive geography-related variability study, which involved determining the actual contribution of each EBV gene to the geographic segregation of the entire genome. Moreover, to the best of our knowledge, we established for the first time the evolution rate for the entire EBV genome based on a host–virus codivergence-independent assumption and assessed their evolution rates on a gene-by-gene basis, which were related to the encoded protein function. Considering the evolution of dsDNA viruses with a codivergence-independent approach may lay the basis for future research on EBV evolution. The exhaustive bioinformatic analysis performed on this new dataset allowed us to draw a novel set of conclusions regarding the genome evolution of EBV.

## 1. Introduction

*Human gammaherpesvirus 4*, commonly referred to as Epstein–Barr virus (EBV), is the type species member of the *Lymphocryptovirus* genus, within the *Herpesviridae* family. After primary infection, EBV establishes life-long latency in memory B-lymphocytes in the human host and is present in over 90% of the world’s population. This aspect has rendered EBV as one of the most successful human viruses, which is assumed to have been co-evolving with its host since the origins of mankind [1]. In developing regions, primary infection often occurs during early childhood and is not usually associated with clinical symptoms, although mild cases of infectious mononucleosis (IM) may occur. On the other hand, in developed regions, where primary infection is usually delayed until adolescence or early childhood, severe cases of IM are more frequent [2]. Although latency does not represent a significant risk for immunocompetent individuals, a co-factor involvement has been suggested in the development of neoplastic pathologies, such as Burkitt’s lymphoma (BL), Hodgkin’s lymphoma (HL), post-transplant lymphoproliferative disorder (PTLD), and cancers of epithelial origin, including nasopharyngeal carcinoma (NPC) and some cases of gastric carcinomas [3].

Based on sequence variation located in the EBNA2 gene and the EBNA3 gene family, EBV is broadly classified as EBV1 and EBV2. Further research at the genetic level has described an association of specific variants with an augmented occurrence in tumoral samples, hence suggesting the possibility of an increased oncogenic potential [4,5]. However, the role of EBV in the etiology and progression of these malignant processes is still not fully understood. The prevalence of EBV-associated malignancies, the percentage of EBV-associated cases, and the viral variants vary worldwide between geographical regions, which suggests the possibility of neoplastic-related EBV variants in different geographical regions, perhaps acting in synergy with genetic or environmental factors [6].

In recent years, many studies from different parts of the world have addressed the analysis of specific signature genes in the quest for viral variants strongly linked to different malignant pathologies [7,8,9]. However, identifying EBV gene variants in tumor samples requires a better understanding of them in the context of the entire genome, so that potentially significant mutations can be distinguished from natural variation.

In recent years, with the advent of next-generation sequencing (NGS) methods, it has been possible to sequence the entire EBV genome, and to date more than 1000 complete genomic sequences isolated from different pathologies and geographical regions are available in GenBank. However, most of these sequences come from Asian isolates, while South American isolates are deeply underrepresented and merely account for an approximate 2% of complete EBV genomic sequences available in the GenBank database.

Hence, we expanded the available data on EBV from Argentina and performed a comprehensive evolutionary analysis of the virus from several regions of the world, which allowed us to study its evolution history on a complete genome scale and on a gene-by-gene basis.

## 2. Methods

### 2.1. Ethics Statement

The hospital’s ethics committee reviewed and approved this study (CEI Nº 17.25), which is in accordance with the human experimentation guidelines of our institution and with the Helsinki Declaration of 1975, as revised in 1983. Clinical samples from patients with EBV infection were anonymized prior to this study. Written informed consent was obtained from all the of patient’s parents or tutors.

### 2.2. Patients and Samples

This study included samples from 15 pediatric patients with EBV-related diseases from Argentina: (i) three children with infectious mononucleosis (IM), with a median age of 4 years (range, 1 to 17 years), 66% male; (ii) a 9-year-old male child with secondary immunodeficiency following liver transplant; (iii) eleven patients with EBV-positive lymphomas (6 Hodgkin and 5 non-Hodgkin), with a median age of 9 years (range, 3 to 18 years), 55% male.

The IM cases were identified on clinical grounds and confirmed by indirect immunofluorescent assay (IFA) for the detection of IgM antibodies against virus capsid antigen (VCA) on commercial EBV-VCA antigen substrate slides (MBL BION, EB-5012) according to the manufacturer’s instructions. The liver transplant patient was followed-up with periodic monitoring of viral load at our institution. Here, 6 mL of EDTA–peripheral blood and a pharyngeal secretion sample were obtained from patients with IM and from the liver transplanted child.

The association between the lymphomas and EBV was examined on previously formalin-fixed and paraffin-embedded tissue sections of the same tumor biopsy using the PNA in situ hybridization (ISH) kit (Dako, K5201), together with a PNA probe to detect Epstein–Barr-encoded RNAs (EBERs) (Dako, Y5200), according to the manufacturer’s instructions. Cases were rendered as EBV-associated when specific staining was observed in the nucleus of tumor cells without staining in infiltrating lymphocytes. In addition, fresh frozen tumor tissue biopsies were obtained from our institutional tumor repository.

### 2.3. DNA Extraction

Total DNA was purified from Ficoll-isolated peripheral blood mononuclear cells (PBMCs), pharyngeal secretions, and fresh-frozen tumor biopsies with the QIAamp DNA minikit (QIAGEN, 51304), according to the manufacturer’s instructions.

### 2.4. Viral Load Assays

The EBV genome copy number was measured in all samples using a real time quantitative PCR (qPCR) assay, as previously described [10], on a LightCycler 480 device. A standard curve was performed with 1/10 serial dilutions of an EBNA1-pGEM-T Easy plasmid, a kind gift from Dr. Dolores Fellner, which ranged from 10^7^ to 10^2^ EBNA1 gene copies. Finally, the viral load was calculated as the EBV genome copy number per µg of total genomic DNA. Samples with a viral load greater than 10^6^ copies/ug DNA were selected for library preparation and sequencing. In those cases where PBMCs and pharyngeal secretions were available, we selected the sample with the highest viral load, assuming negligible viral variation between both anatomical compartments, as previously observed [7].

### 2.5. Library Preparation and Illumina Sequencing

Sequencing libraries were constructed with SureSelect QXT Target Enrichment kit (Agilent Technologies, G9683B) and custom-designed EBV RNA bait probes (Agilent Technologies, 5190-4806), according to the manufacturer’s instructions. RNA bait probes were designed and kindly shared by Dr. Daniel P. Depledge [11]. EBV-enriched libraries were sequenced on a NexSeq500 Illumina device, with 300 cycle mid-output (2 × 150 bp) NextSeq Reagent kits v2 (Illumina Corporation, 20024905). FASTQ files of raw sequencing data were deposited in the NCBI database with the Sequence Read Archive (SRA) accession number PRJNA679281 (Supplemental Table S1).

### 2.6. Publicly Available Sequence Data

Raw NGS data were downloaded from the SRA-NCBI database (Supplemental Table S1). Downloaded data belonged to BioProjects numbers PRJNA522388, PRJNA505149, and PRJEB2768.

### 2.7. Bioinformatic Data Processing

The bioinformatic analysis was implemented with a mapping-based customized pipeline designed at the bioinformatics core of our institution. Initial preprocessing steps, including read quality checks and sequence trimming, were performed with fastp software v.0.20.1 [12]. Duplicate removal was assessed with PRINSEQ software v.0.20.4 [13]. Thereafter, reads were mapped to both EBV-type reference genomes (NC_007605.1 for EBV1 and DQ279927.1 for EBV2), making use of Burrows–Wheeler Aligner (BWA) v.0.7.17-r1188 [14], which generates BAM format output files. After this, BAM files were processed using SAMTools v1.9 [15]. This phase uses both available reference genomes and reports the number of variants over EBNA2 and the entire EBNA3 family genes to establish the EBV type. The variant calling step and consensus sequence generation were performed with SAMtools software package v.1.7 (bcftools mileup) [15]. In addition, our pipeline takes into consideration masked repetitive regions, as well as the uncovered ones, in order to avoid biases in the detection procedure of indels.

After the mapping step against both reference genomes, the EBV type was predicted based on the number of variants, considering EBNA2 and the entire EBNA3 family of genes. This typing step uses the VCF file and operates under the assumption that a greater number of genetic variants will arise when mapping against the wrong type reference; on the contrary, a better mapping quality will result when mapping against the correct reference (Supplemental Table S2).

New NGS raw data from samples sequenced in this study, as well as raw data downloaded from NCBI, were analyzed with the same bioinformatic pipeline.

### 2.8. Multiple Sequence Alignment and Evolutionary Tests

Multiple sequence alignment (MSA) was performed with MAFFT v7.310 [16] and curated manually for further gap reductions.

Aligned genomes were assessed for potential recombination signals with RDP4.101 software [17], and those sequences deemed as potentially recombinant genomes were removed from the alignment.

IQ-TREE software v1.6.1 [18] was used to determine the best partition schemes for the MSA and to predict the best evolutionary model for each partition (Supplemental Table S3). Then phylogenetic reconstruction was performed under the maximum likelihood (ML) method and 1000 ultrafast bootstrap iterations were performed. The ML ultrafast bootstrap tree was visualized in FigTree v1.4.3 (http://tree.bio.ed.ac.uk/software/figtree/; accessed on 5 December 2019). Geography-based segregation analysis of EBV genomes was performed with iTOL [19].

The alignment was imported into R software v4.0.2 [20] using the ape package and the SNPs were extracted using the adegenet package [21,22]. Principal components analysis (PCA) and Principal Component Discriminant Analysis (DAPC) were performed with the adegenet package.

The EBV1 reference sequence was compared with sequences belonging to the same geographical region with Consensus Compare tool, kindly provided by Dr. PJ Farrell, to evaluate specific geographical polymorphisms.

### 2.9. Estimation of Evolution Rate

Fifty-five sequences with sampling dates ranging from 1963 to 2019 and from different regions of the world were further selected for evolution rate estimation (10 EBV2 and 45 EBV1). Since this new dataset presented no temporal structure, a previously published evolution rate for the LMP1 gene—obtained from data with a temporal structure—was used for molecular clock calibration [23]. The time to the most recent common ancestor (tMRCA) for LMP1 was initially estimated and used to calibrate the rest of the analyses. Evolutionary models were estimated with IQ-TREE software v1.6.1 (Supplemental Table S4). Bayesian coalescent analysis was implemented in BEAST v1.8.4 [24] using an uncorrelated lognormal relaxed clock model (UCLN) and a Gaussian Markov random field (GMRF) Bayesian skyride demographic model. Analyses were run for 80 to 100 million Markov chain Monte Carlo (MCMC) iterations. Results were visualized in Tracer v-1.7 [25] and convergence was assessed with effective sample sizes higher than 200.

### 2.10. Gene Ontology-Based Clustering

Genes were grouped using an unsupervised procedure that took into consideration three well-distinguished Gene Ontology (GO) domains: Biological Process (BP), Cellular Component (CC), and Molecular Function (MF). First, direct EBV gene annotations were obtained from the Uniprot database (https://www.uniprot.org/, accessed on 15 July 2020) and extended to all its parental relations among each domain. This information was summarized by means of a binary matrix constructed for each GO domain. Then, a gene-to-gene distance matrix for each domain was computed based on the Jaccard index. Over these matrices, the presence of a non-random structure was explored using both the Hopkins statistics and Visual Assessment of Cluster Tendency (VAT) in clustertend v1.4 [26] and factoextra v.1.0.7 [27] R packages. Finally, a K-means algorithm was used to undertake gene groups for each GO domain. The optimal number of partitions (K) was initially explored through three quantitative methods (Elow, gap statics, and average silhouette) and then refined manually by merging clusters with redundant biological interpretation.

### 2.11. Statistical Analysis

Statistical analysis was performed using the computing environment R version 4.0.2. Normality and homoscedasticity were evaluated using Shapiro–Wilk and Bartlestt tests, respectively. Groups that met these principles were analyzed with Student’s t-test and groups that didn’t meet these criteria were compared with Wilcoxon or Kruskal–Wallis tests, as appropriate. All *P*-values were adjusted using the Bonferroni test.

## 3. Results

### 3.1. A Call for a Unified Raw Data Sequence Analysis

For the first time in our country, fifteen EBV-associated samples from pediatric Argentinean subjects were sequenced by NGS methods and were further analyzed with an automated and customized pipeline for viral data. To compare the variability of our genomes in the context of EBV sequences from other geographies, 199 publicly available raw NGS data were downloaded from SRA-NCBI database and re-analyzed together with our raw data with our new custom EBV bioinformatics pipeline (see methods). Using this procedure, a total of 189 samples were classified as having type 1 EBNA2 and EBNA3s genes, 21 were type 2 EBNA2 and EBNA3s, while only 4 were classified as recombinant genomes with type 1 EBNA2 and type2 EBNA3s. Regarding the 15 new Argentine sequences, 9 were classified as EBV1 and 6 were classified as EBV2 (Supplemental Figure S1).

**Figure 1:**
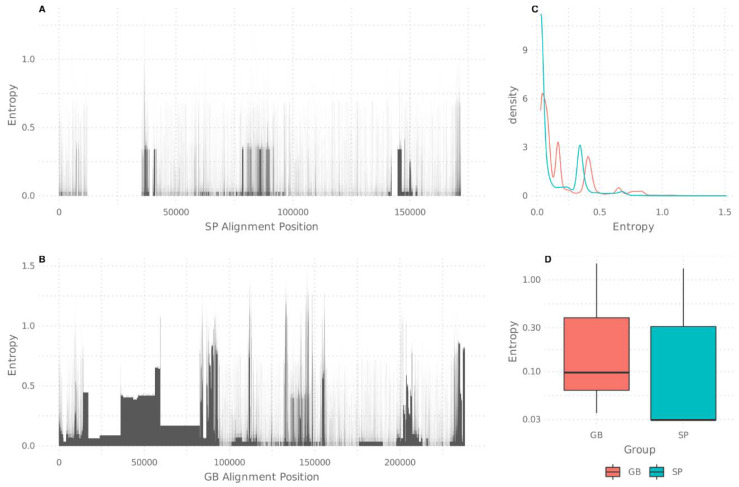
Alignment entropy analysis: (**A**) entropic positions in the single pipeline (SP)-generated alignment; (**B**) entropic positions in the alignment directly generated with GenBank (GB)-downloaded sequences, for which highly entropic positions are depicted as black spikes or blocks; (**C**) entropy density chart for both SP and GB alignments, considering all alignment positions; (**D**) entropy comparison considering only positions with entropy >0, depicted in box plots.

Following viral typing, consensus sequences were constructed and aligned. Two different settings were explored: an MSA constructed with direct consensus sequences downloaded from GenBank (GB) and an MSA constructed with all sequences re-analyzed with a single pipeline (SP). Interestingly, SP sequences uniformly analyzed from fastq files produced a more reliable alignment than directly aligned consensus sequences from GenBank. In the first place, GB consensus sequences produced a longer alignment due to multiple gap insertions, compensating for methodological discrepancies that arise when different pipelines are used (Figure 1A,B). Moreover, while SP alignment preserves more than 82% of its positions perfectly (zero entropy), GB consensus keeps only 28% of its extent perfectly aligned (Fisher’s exact test: OR = 11.8 IC_95%_ (11.3–11.7), *p*-value < 2.2 × 10^−16^). Concerning variability and SNPs, the SP alignment reported 30,824 SNPs, while the GB consensus alignment reported 169,845 SNPs, most of which were located in repetitive regions; this made the GB alignment far more variable (Fisher’s exact test: OR = 0.086 IC95% (0.085–0.088), *p*-value < 2.2 × 10^−16^). The latter case lacked biological plausibility, since the double-stranded DNA genome and the known stability of EBV are not compatible with the idea of having 70% variable regions along its genome. On the other hand, a major lack of homogeneity was observed with the GB consensus procedure, which contains a significant number of entropic positions and a right-shifted entropy distribution in comparison with SP alignment (Figure 1C). When only analyzing variable positions in both alignments (Entropy >0), significant differences were found between distributions (Wilcoxon’s test, *p*-value < 2.2 × 10^−16^) (Figure 1D). Overall, these results point out the drawbacks that arise when GenBank consensus sequences are directly considered to build an MSA and highlight the need for a unified process starting from raw fastq files.

### 3.2. Phylogenetic Relations, Evolution, and Geographic Segregation

**Figure 2:**
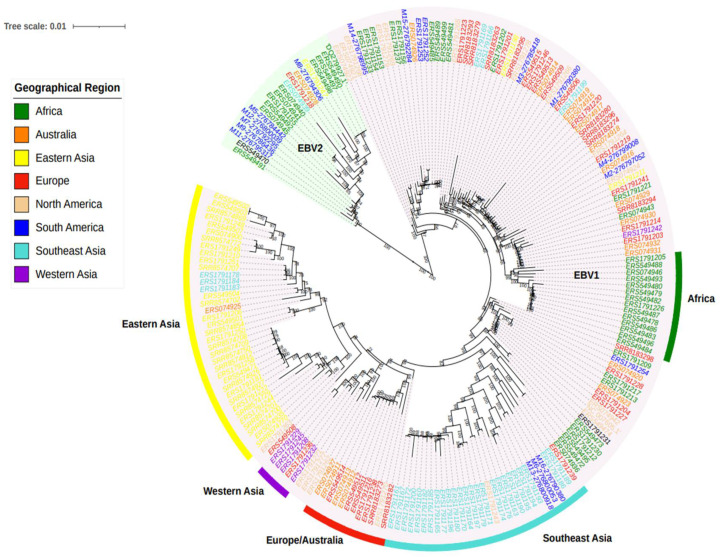
Phylogenetic reconstruction of 214 complete EBV genomes from different geographies. Phylogenetic tree constructed under the maximum likelihood method and 1000 ultrafast bootstrap resampling iterations. Only values over 70 are shown. The green shaded clade contains the EBV2 sequences and the pink shaded clade is EBV1. The color of each sequence represents the geographical region of the viral isolate. One sequence without a reliable origin of isolation is labeled in black (omitted in further analyses). The five subclades were highlighted using different externally colored bars. South American sequences correspond to isolates sequenced in the present study or were previously sequenced isolates from Argentina.

Following the construction of the new consensus sequences and removal of recombinant genomes, the phylogenetic relationship between the Argentinean and worldwide genomes was inferred. As expected, the phylogenetic tree depicted two major clusters, EBV1 and EBV2. Within the EBV1 clade, two well-supported major subclusters were observed, with some inner groups with defined geographic structures. The first subcluster was mainly composed of Asian sequences, while the second subcluster was cosmopolitan, including intermingled sequences from Europe, Australia, and the Americas, with some sequences from Argentina, as well as a supported inner group of African sequences. Furthermore, the first subcluster showed four subgroups: a small cluster formed by sequences from Western Asia (India, Saudi Arabia), two larger groups representing Eastern Asia (China, Japan, South Korea, Taiwan, and Hong Kong) and Southeast Asia (Indonesia, Papua New Guinea, and Singapore), and a group of sequences from Europe and Australia. In addition, three sequences from Argentina were associated with viruses from Southeast Asia (Figure 2). Regarding the EBV2 clade, most of the sequences were from Africa and Argentina, with minor representation of other geographic regions; however, the scarce number of EBV2 genomes prevented further statistical confidence; hence, subsequent geographic analysis was performed considering EBV1 genomes only.

**Figure 3:**
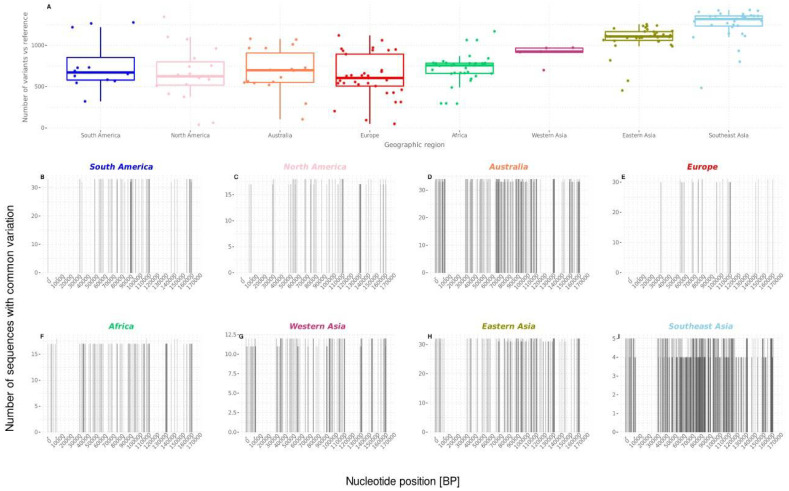
Quantification of EBV1 genomic variation regarding geographic origin: (**A**) quantification and comparison of the amounts of variants from different regions, with the results depicted in box plots; *(***B**–**I***)* positioning of common variants against the EBV1 reference along the genome for each geographical region.

In order to statistically assess the number of variants within each region, variants were obtained from the VCF file by mapping the reads against the EBV1 reference sequence, which were then geographically stratified (ERS1791231 was omitted). This geographical variability was statistically significant, since sequences from Africa, Europe, Australia, North America, and South America presented similar amounts of variants when compared to the reference. On the other hand, Asian genomes (South East Asia, Eastern Asia, and Western Asia) contained significantly more variants when compared to the reference genome (Kruskal–Wallis test, *p* < 2.2 × 10^−16^), while at the same time presented the least intra-group variability, as indicated by the height of the box plot (Figure 3A) (Supplemental Table S5).

To further elucidate the positioning of common variants within the EBV1 genome for each geographical region, independent alignments of geographically tagged sequences were generated and compared to the reference sequence using the custom-built Consensus Compare tool (Figure 3B–I). The sequence variation was not homogeneously distributed along the genome in the different geographies. For instance, Asian sequences presented the most variable pattern of common variation along the genome; the region between 120,000 pb–140,000 pb, which codes for late lytic genes (BDLF2, BDLF1, BcLF1, BcRF1, BTRF1, BXLF2, BXLF1, BVRF1, BVLF1, BVLF2, BdRF1, and BILF2), depicted variants in the South Asian, Eastern Asian, and Western Asian sequences that were not present in the other geographical groups.

**Figure 4:**
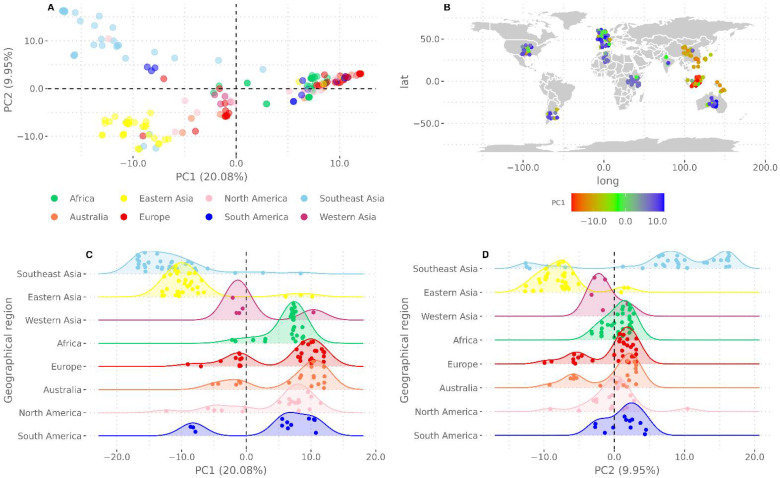
Principal component analysis: (**A**) PCA of EBV1 sequences showing PC1 and PC2, whereby sequences are colored according to their geographic origin; (**B**) geographical distribution of the sequences, whereby the color intensity in the heat map scale represents the segregation potential of PC1; (**C**,**D**) quantification of the differences in PCA distribution (**C**) for PC1 and (**D**) for PC2.

**Figure 5:**
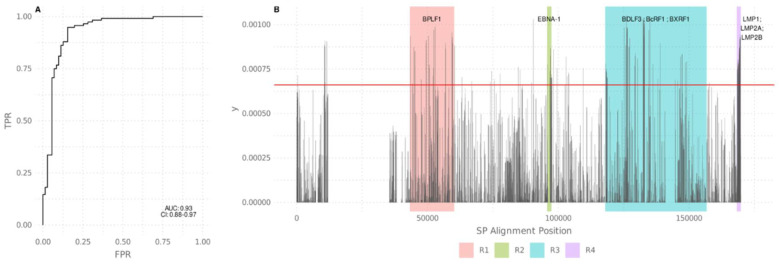
Discrimination analysis of principal components (DAPC): *(***A***)* ROC curve depicts the segregation potential of the sequences shown in Table 1; (**B**) DAPC variance contribution of each site (SNP) along the EBV1 genome. The red horizontal line indicates the threshold (0.00066) above which structural SNPs were identified. Colored boxes delimit the 4 informative coding regions of the genome. The most relevant genes within each region are indicated on the top of each box.

PCA was conducted as a complementary analysis to the phylogenetic tree and in order to further assess the geographic structure in the EBV1 group. As shown in Figure 4A,B, PC1 (20.08%) mainly segregated the Asian genomes from the rest of the global sequences, while PC2 (9.95%) allowed for the further discrimination of the Asian group into the three previously described subgroups; furthermore, both PC1 and PC2 distributions differed significantly among geographies (Kruskal–Wallis test, *p*-values: 8.58 × 10^−22^ and 5.88 × 10^−17^, respectively). Following this result, a pairwise Wilcoxon’s test was performed among all geographic regions. This analysis quantified the observation that the Eastern Asian, Western Asian, Southeast Asian, cosmopolitan genomes differed significantly in their first and second PCA distributions (Figure 4C,D, Supplemental Table S6). According to the observed results, which supported the differential segregation of Asian EBV genomes from those from other continents, a discriminant analysis of principal components (DAPC) was performed with the aim of identifying those genome regions that drive the genetic divergence between both groups [22]. The number of PCs retained was defined according to the proportion of successful reassignments corrected for the number of retained PCs (alpha score); this procedure was conducted for both groups in order to improve the discrimination of the groups and to avoid overfitting. According to this criterion, only DAPC1 was retained, which was sufficient to summarize the gene diversity between the Asian continent and the rest of the geographical regions. Hence, the probability rates of allocating Asian and non-Asian genomes to the correct geographic origin were 84.5% and 89.65%, respectively. Then, an ROC curve was constructed to further estimate the potential of this classifier system, which gave an AUC of 0.93 (0.88–0.97) (Figure 5A). After this, structural SNPs were identified by means of the snpzip function of the adegenet package [22], which allowed for the identification of the four informative coding regions that were relevant in the segregation of Asian and cosmopolitan non-Asian clades (Figure 5B). The first region was located between 43,281 to 60,240 bp with respect to the reference genome; variants located in this region were mainly concentrated in the BPLF1 gene. The second region was located between 95,719 to 97,415 bp; variants in this region were concentrated in the EBNA-1 gene. The third group was located between 117,949 to 156,739 bp; although a large number of lytic genes were found in this region, variants were mainly concentrated in BDLF3, BcRF1, and BXRF1 genes. Finally, the fourth region was located in the terminal region of the genome (168,236 to the end with respect to the reference), where the genes that encode for the oncogenic proteins LMP1, LMP2A, and LMP2B are located. Additionally, isolated variants that also contributed to this differentiation were found in other regions of the genome with a lesser frequency (Supplemental Table S7).

### 3.3. Genomic Evolutionary Rates

**Figure 6:**
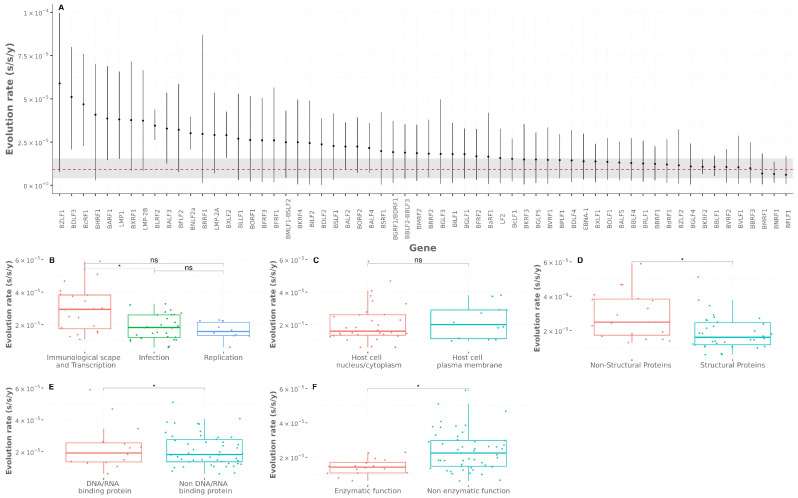
Analysis of evolutionary rates: (**A**) evolution rates for 60 analyzed genes, where dots depict mean evolution rates, error bars indicate 95% HPDI (highest posterior density interval), the red dotted line depicts the genome’s mean evolution rate, and the gray horizontal stripe denotes the 95% HPDI for the genome´s mean evolution rate; (**B**–**F**) comparison of the genes’ evolution rates among different groups contained in the biological categories (gene names listed in Table 1). Results are depicted in box plots. Asterisks denote statistical significance. Adjusted *p*-value ˂ 0.05 (*); adjusted *p*-value > 0.05 (ns).

Given that each gene contributed differently to geographic segregation, we sought to calculate the evolution rates of the entire genome and of each individual gene (Figure 6A, Supplemental Table S4). In total, 40 of the 60 analyzed genes exhibited evolutionary rates above the calculated mean for the entire genome (mean: 9.09 × 10^−6^ s/s/y; 95% HPD interval: (4.08 × 10^−6^, 1.56 × 10^−5^)). In particular, BZLF1, BDLF3, and BcRF1 were amongst those genes with the highest evolutionary rates; interestingly, most of them are involved in viral immune escape, as reported in the GO database. On the other hand, a total of 20 genes exhibited a similar evolutionary rate to that of the entire genome, with BFLF1, BMRF1, and BNRF1 being amongst those with the lowest evolutionary rates. As a whole, these latter genes are mainly involved in viral processes such as DNA replication and viral particle assembly.

For a comprehensive analysis of the genes’ evolutionary rates, the EBV genes were grouped based on three genetic domains, as defined in the GO database, by means of an unsupervised analysis. The groups were obtained using the K-means method and were further refined and merged manually considering their annotated function in order to avoid redundancies and produce better fits for the groups. In addition to those gene domains defined by the unsupervised analysis, two additional categories were defined on the basis of biological criteria, namely the “viral component” and “enzymatic function”. The resulting groups are presented in Table 1.

Biological Process: Three groups were obtained after the unsupervised analysis using GO Biological Process domain. The first group included genes involved in immunological escape. The second group contained genes implicated in the interaction between viral and host cells and the fusion of the viral and cell membranes. Finally, the third group included only genes necessary for DNA viral replication.

Cellular Component: Again, three groups were obtained within the GO Cellular Component domain. The first group was defined by integral proteins located in the host cell membrane or in the virion membrane. The second group contained proteins that exert their action on the nucleus and cytoplasm of the host cell. The last group was defined by proteins present on the extracellular space; however, this group was not further considered in the evolutionary rate analysis, since it was composed of only two genes.

Viral Component: Additionally, two groups were manually curated from the GO Component Cellular domain—genes coding for structural and non-structural proteins.

DNA/RNA binding protein: Only 31 genes were annotated with GO Molecular Function domain; two gene product groups were categorized, those with the ability to bind DNA or RNA and without the ability to bind DNA or RNA.

Enzymatic Function: Additionally, two groups containing enzymatic and non-enzymatic functions were manually curated using the GO Molecular Function domain.

The numbers of genes in the mentioned groups were further enlarged with genes with an annotated function in GenBank.

Using all of the previously defined groups, the calculated evolutionary rate for each EBV gene was fitted into these categorical groups and the groups’ mean evolutionary rates were compared in pairs.

First, we compared the evolution rates among groups contained in the GO Biological Process category. Genes involved in viral immunological escape presented higher evolutionary rates than genes implicated in the entry and exit of the viral particle (Wilcoxon’s test, Bonferroni correction, *p* = 0.022; Figure 6B) and also tended to be greater than genes associated with DNA viral replication (Wilcoxon’s test, Bonferroni correction, *p* = 0.074). No differences were found between mean evolutionary rates from genes needed for DNA biosynthesis and genes involved in viral entry and assembly (Wilcoxon’s test, Bonferroni correction, *p* = 1).

Next, we assessed differences in evolutionary rates of genes in the GO Cellular Component domain. The mutation rates of gene products located in cellular membranes did not differ from those of gene products localized in the cell nucleus (Wilcoxon’s test, Bonferroni correction, *p* = 1; Figure 6C). Within the GO Viral Components group, those genes that code for non-structural proteins had higher evolutionary rate than genes coding for structural proteins (Wilcoxon’s test, Bonferroni correction, *p* = 0.024; Figure 6D).

When comparing evolutionary rates of genes in the DNA/RNA binding protein group, these evolutionary rates did not differ between gene products with the ability to bind DNA or RNA and those with no such ability (Wilcoxon’s test, Bonferroni correction, *p* = 1; Figure 6E). Finally, the evolution rates for those genes with enzymatic function were smaller than those not coding for enzymatic proteins (Wilcoxon’s test, Bonferroni correction, *p* = 0.014; Figure 6F).

Overall, the results of this analysis, which covers the gene-by-gene evolution rate calculus with its relation to their biological functions, support the well-established notion that genes under constant immune pressure will have higher evolution rates in order to evade immune surveillance.

## 4. Discussion

In this paper, we sequenced 15 complete EBV genomes from pediatric patients from Argentina and analyzed their phylogenetic relation and variability pattern in the context of other complete genomes from different parts of the globe. Furthermore, we assessed the evolution rates of both the entire genome and at a gene-by-gene level while exploring the biological significance of their evolution rates in relation to their functionality or localization of the gene product to the cell or viral particle.

Other research groups have previously generated up to 1000 complete EBV genome sequences from different geographic locations [28,29]; however, each group analyzed their datasets in an independent way, without any standardized criteria. This is not a problem for each individual analysis but becomes a major concern when integrating all independently generated consensus sequences for further analysis. There is no unique gold-standard bioinformatic pipeline to obtain the final EBV genome consensus sequence, something of particular importance given the amount of repetitive regions in EBV genome and given that different research groups would obtain different consensus genomes even if analyzing the same dataset. For instance, systematic differences were observed in the way that each group processed repetitive regions of the genome, deletions sites, or poorly covered regions. Some bioinformatic strategies replaced the lack of a good quality sequence by the reference sequence, while others replaced the lack of a good quality sequence with gaps, with unknown nucleotides (N), or even by cutting off that region, all of which will have different meanings in post-phylogenetic analysis. Such discrepancies become most relevant when comparing sequences from different sources, as we did in this study. In order to produce higher quality results, we demonstrated the importance of analyzing the raw fastq data files using the same pipeline instead of aligning the consensus sequences directly downloaded from NCBI. In this scenario, our new pipeline took special consideration of these issues by masking conflictive regions with N nucleotides and conserving small insertions and deletions (Indels), since it retained this information to produce the final consensus genome.

The sequencing of complete EBV genomes from Argentina and their integrated phylogenetic analysis with genomes from other regions of the world revealed the presence of two groups supported by a high bootstrap value, with a small group formed by EBV2 sequences and a much larger group composed of EBV1 sequences. From the full data set, 18 sequences were from Argentina, 15 were newly sequenced, and 3 were downloaded from NCBI. These sequences were found in both EBV1 and EBV2 groups and were interspersed among sequences from different geographic locations, indicating their independent origins. This study enlarges the amount of fully characterized EBV genomes from our region, especially from Argentina, which were until now underrepresented in the NCBI database; moreover, reporting new EBV2 genomes is of particular interest, since EBV2 genomes from South America are even scarcer [28,29].

After restricting the analysis to EBV1 genomes, a geographical structure arose for this viral type from the phylogenetic and principal component analysis. In the former case, bootstrap supports allowed for the discrimination of the EBV1 clade into a great Asian clade, which was further differentiated into four smaller subclades (Western Asia, Eastern Asia, Southeast Asia, and Europe–Australia). On the other hand, a cosmopolitan clade contained genomes from Africa, Europe, Australia, and the Americas. In the latter case, PC1 mainly separated the Asian sequences from the rest of the geographies but was also able to further discriminate the Asian group into a Southeast Asian group and another group composed of Eastern and Western Asian genomes. Furthermore, PC2 further discriminated the Southeast Asian sequences from the rest of Asia. This geographical structure was in accordance with previous observations [29,30], even after the inclusion of our 15 Argentinean sequences, indicating the high segregation potential of the Asian sequences. Another interpretation of this fact could be the high resemblance of American sequences to those from Africa, Australia, and Europe. This latter interpretation could be plausible given the imperialist history of humanity and historical movements of humans, since the American, African, and Australian continents have undergone large-scale population changes from European colonialism and migration [31]. In particular, an estimated 1.5 million European migrants arrived to the Americas along with the introduction of an estimated 7 million African slaves between the 15th and 18th centuries [32]. It is worth mentioning that having possible recombinant sequences removed from the alignment strengthens our observations, since recombinant genomes could distort the topology of the tree, as demonstrated by Zanella et al. [33]. In this way, we produced a reliable phylogenetic reconstruction with the entire genome sequence and obtained similar results regarding geographic variants, as previously described [28].

Considering Asian sequences presented higher variability with respect to the EBV wild-type reference than those from other regions and that those variants were distributed along the entire genome rather than in a single gene, these factors could support the notion of demographic events during ancestral human migration. In addition, the low internal diversity observed among Asian sequences might suggest the existence of a bottleneck event. Remarkably, this evolutionary pattern was also observed for other herpesviruses, namely HSV1 and HHV8 [34,35], as well as for other viruses [36]. Overall, these results support the idea that one EBV reference genome does not fully represent wild-type EBV for all geographies.

Previously, using first-generation gene sequencing techniques for certain latent genes, it was proven that they could contribute to the differential segregation of Asiatic sequences [9,37]. Nowadays, massive sequencing techniques allow us to identify variants that prevail in each geography and their distribution over a whole genomic scale, enabling us to detect those genes mainly contributing to this clustering. Given the prior observations in the phylogenetic tree and PCA, DACP was implemented to disclose which genomic positions contributed to the segregation of the two main groups, namely the Asian and the cosmopolitan non-Asian clades. To the best of our knowledge, this is the first time that the contribution of each single gene or group of genes has been assessed in the context of the entire EBV1 genome in this way. The analysis revealed four main genomic regions with geographic segregation power. Variants in the first genomic region were mainly concentrated in the BPLF1 gene. A previous study reported a large content of non-synonymous variants in this gene when assessing gastric carcinoma and control samples; however, no association between these variants and their oncogenic potential has been disclosed until now [38]. Nonetheless, the authors suggested that the BPLF1 gene could be influenced by positive selection, although the variability of this gene in the geographical context had not yet been reported. Variants present in the second group were identified in the terminal region of the EBNA-1 gene. This gene has been thoroughly sequenced in order to study its association with malignant pathologies and geographies and has proven to be a good molecular marker of geographic origin, based on amino acid position 487 in the C-terminal region of the protein [9,39,40]. Our present results are in accordance with these previous observations that the EBNA1 gene is a strong geographical indicator.

Concerning the region located between positions 120,000 and 140,000 of the reference genome, the main geographical segregating SNPs were located in the BDLF3, BcRF1, and BXRF1 genes, which are late-phase genes; however, as far as we know, there are no reports concerning these gene variants with respect to their geographic origin.

Finally, the last region with geographic segregation potential was mapped to the latent membrane proteins, LMP1, LMP2A, and LMP2B. Remarkably, the geographical variability of these genes had been previously well characterized with different genetic approaches, but not when considering the segregation contribution of each individual gene when influenced by the other genes in the genome [9,39]. Moreover, our group previously characterized a distinct LMP1 variant with preferential circulation in our country [23], a fact that highlights the actual value of enlarging the proportion of complete genomes from our country in order to overcome the current scarcity of data from South America.

In this study, the evolution rate of each gene and the rate for the entire genome were calculated in order to characterize the genes with higher mutational rates. Given that the EBV genome is made up of a large double-stranded DNA molecule (dsDNA) of approximately 172 kpb and has a DNA polymerase with reliable proof-reading activity, a high degree of conservation was expected [41,42]. Such analysis requires a large number of sequences covering an ample scale of time in order to achieve an appropriate temporal structure for phylodynamic analysis. From the original data set, only 55 sequences had information on the isolation date, this being the main limitation to understanding the evolution over time of each of the EBV genes. There are two possible strategies to overcome this limitation—one of them is to assume the co-evolution of the EBV genome and the host genome, while the second possible strategy is to calibrate the analysis with an evolution rate reported in the bibliography for a particular gene of the same virus or of an evolutionarily close virus, estimating the age of the common ancestor and then calibrating the remaining analyses with these data [43]. In particular, our analysis was performed using the second strategy, because with the assumption of a virus-host co-evolution, the viral evolutionary rate would be forced to be on the same evolutionary scale as that of its host. On the contrary, the second analysis strategy makes it possible to independently estimate the viral evolutionary rate. In our study, which made use of 55 EBV isolates from different geographic regions, we estimated a substitution rate for the entire EBV genome (9.09 × 10^−6^ s/s/y), which was two orders of magnitude higher than that of the human host (0.5 × 10^−9^ s/s/y) [44] and similar to that of other double-stranded DNA viruses, including herpesviruses [36]. This result suggests that EBV co-evolving together with humans is not necessarily the most appropriate assumption for this virus, in accordance with a previous observation for HSV-1 and VZV [36].

In addition, the evolutionary rates for most individual genes were evaluated, with BZLF1, BcRF1, BDLF3, BXRF1 and LMP1 being among those that showed the highest evolution rates. Notably, these genes were also the same genes that contributed most to both viral type segregation (after EBNA2 and EBNA3s) and geographic segregation.

Finally, we analyzed the evolutionary rates in terms of functional gene groups, which were constructed according to the GO domains. Not surprisingly, our analysis showed that genes coding for viral immunoescape-related proteins have accumulated higher number of variants over time, a selection characteristic that may have provided the virus with the ability to latently persist for a lifetime in the infected host. As expected, genes coding for enzymes showed lower evolutionary rates, which translated into higher levels of conservation given the necessity for functional enzymes in order for EBV to undertake sporadic reactivation and replication cycles. In addition, it is worth mentioning that genes coding for structural proteins (proteins packed in the viral particle) showed evolutionary rates significantly lower than non-structural ones, another characteristic of evolutionary importance given that structural proteins will determine the success of early infection stages. Moreover, this fact was consistent with the previous observation, since most gene products involved in immunoescape are indeed non-structural proteins.

Given that circulating EBV genomes in South America are underrepresented, this work constitutes a valuable contribution to a better understanding of the relationship between viral variability and geography-related demographic events. Additionally, a large number of EBV2 sequences have been added but even so the number of sequenced genomes of this viral type is still small, making statistical analyses implausible and meaning it is not possible to reach a reliable conclusion. Past events such as the arrival of Europeans to America and more recent events such as human migration could explain the relationships observed in the phylogenetic analysis.

In summary, this study contributes to overcoming the scarcity of complete EBV genomes from South America and represents the most comprehensive geography-related variability study, which involved determining the actual contribution of each EBV gene to the geographic segregation of the entire genome. Moreover, to the best of our knowledge, we established for the first time the evolution rate for the entire EBV genome and statistically demonstrated that evolution rates, on a gene-by-gene basis, are related to the encoded protein function. Considering the evolution of dsDNA viruses with a codivergence-independent approach may lay the basis for future research on EBV evolution. Finally, this work also expands the sampling time lapse of available complete genomes derived from different EBV-related conditions, a matter that until today still prevents detailed phylogeographic analysis.

## Figures and Tables

**Table 1:** The table describes the groups as defined by the unsupervised analysis from the Gene Ontology database and the groups created based on their biological significance and those genes belonging to each group.

**Biological Process**
Viral immunological escape and transcription LMP1, LMP2A, LMP2B, BGLF4, BZLF1, EBNA1, BMLF1-BSLF2, BILF1, BLRF2, BNLF2a, BGLF5, LF2, BCRF1, BHRF1, BRLF1, BcRF1, BARF1, BLDF3, BILF2, BRRF1
Viral infective BBRF3, BGLF3, BZLF2, BcLF1, BXLF2, BSRF1, BDLF1, BdRF1, BFLF1, BPLF1, BFRF3, BDLF2, BLLF1, BFRF1, BFLF2, BKRF2M BVRF1, BMRF2, BGLF1, BBRF2, BALF3, BGRF1/BDRF1, BALF4, BBRF1, BOLF1, BNRF1, BORF1, BVRF2, BBLF1
Viral replicative BMRF1, BSLF1, BBLF2, BALF2, BALF5, BXLF1, BaRF1, BORF2, BBLF4, BKRF3
**Cellular Component**
Host cell plasma membrane BLLF1, BALF4, BXLF2, BKRF2, BMRF2, BBRF3, BBLF1, BZLF2, BILF1, LMP1, LMP2a, LMP2b
Host cell nucleus/cytoplasm BMRF1, BGLF4M BPLF1, BFLF1, BLRF2, BXRF1, BGLF1, BBRF2, BSRF1, BDLF1, BORF1, BFRF3, BVRF1, BcLF1, BVRF2, BNRF1, BOLF1, BXLF1, BALF2M BBRF1, EBNA1, BZLF1, BHRF1, BcRF1, BSLFS/BMLF1, BALF5, BGLF5, BBLF4, BALF3, BKRF3, BSLF1, BaRF1
Extracellular space BCRF1, BARF1
**Viral Component**
Structural proteins BLLF1, BALF4, BXLF2, BKRF2, BMRF2, BBRF3, BBLF1, BZLF2, BDLF3, BDLF2, BFRF2, BKRF4, BdRF1, BGLF4, BMRF1, BPLF1, BFLF1, BLRF2, BXRF1, BGLF1, BBRF2, BSRF1, BDLF1, BORF1, BFRF3, BVRF1, BcLF1, BVRF2, BNRF1, BOLF1, BXLF1, BALF2, BBRF1, BALF5
Non structural proteins LMP1, LMP2a, LMP2b, BILF1, BILF2, EBNA1, BZLF1, BcRF1, BCRF1, BARF1, BHRF1, BSLF2-BMLF1, BGLF5, BBLF4, BKRF3, BSLF1, BaRF1
**Enzymatic Function**
Enzymatic function BGLF5, BGLF4, BGRF1/BDRF1, BPLF1, BKRF2, BVRF2, BSLF1, BORF2, BBLF2, BaRF1, BKRF3, BXLF1, BALF5, BBLF4, BLLF3, BMRF1
Non enzymatic function BZLF1, BDLF3, BcRF1, BHRF1, BARF1, LMP1, LMP2a, LMP2b, BLRF2, BNLF2a, BRRF1, BMLF1-BSLF2, BILF2, BGLF3, BILF1, BFRF2, BDLF4, EBNA1, BRLF1, BVLF1, BXRF1, BALF3, BFLF2, BXLF2, BDLF1, BLLF1, BORF1, BFRF1, BFRF3, BKRF4, BDLF2, BALF4, BSRF1, BMRF2, BBRF2, BGLF1, BcLF1, BVRF1, BOLF1, BBRF1, BdRF1, BZLF2, BBLF1, BBRF3, BNRF1, BFLF1, BALF2
**DNA Binding Protein**
DNA/RNA binding protein BGLF5, BGRF1/BDRF1, BKRF2, BSLF1, BBLF2M BALF5, BBLF4, BMRF1, BZLF1, BcRF1, BLRF1, BMLF1-BSRLF2, EBNA1, BORF1, BALF2
Non DNA/RNA binding protein BDLF3, BHRF1, BARF1, LMP1, LMP2a, LMP2b, BNLF2a, BRRF1, BILF2, BGLF3, BILF1, BFRF2, BDLF4, BRLF1, BVLF1, BXRF1, BALF3, BFLF2, BXLF2, BDLF1, BLLF1, BFRF1, BFRF3, BKRF4, BDLF2, BALF4, BSRF1, BMRF2, BBRF2, BGLF1, BcLF1, BVRF1, BOLF1, BBRF1, BdRF1, BZLF2, BBLF1, BBRF3, BNRF1, BFLF1, BGLF4, BPLF1, BVRF2, BORF2, BaRF1, BKRF3, BXLF1, BLLF3

## Data Availability

NGS data generated in this project is available under Bioproject nº PRJNA679281.

## Supplementary Materials

The following are available online at https://www.mdpi.com/article/10.3390/v13061172/s1, Figure S1: EBV typing, Table S1: Sequences description, Tabla S2: EBV variants description, Table S3: Evolution models, Table S4: Genes evolution model, Table S5: Geographic variation statistics, Table S6: Geographic variation-principal component analysis, Table S7: Discriminant analysis of principal components.
