# Supplementary figures and images for: Comprehensive Evolutionary Analysis of Complete Epstein–Barr Virus Genomes from Argentina and Other Geographies
Source: Viruses. 2021 Jun 18;13(6):1172. doi: 10.3390/v13061172 (PMC8235469; doi:10.3390/v13061172)

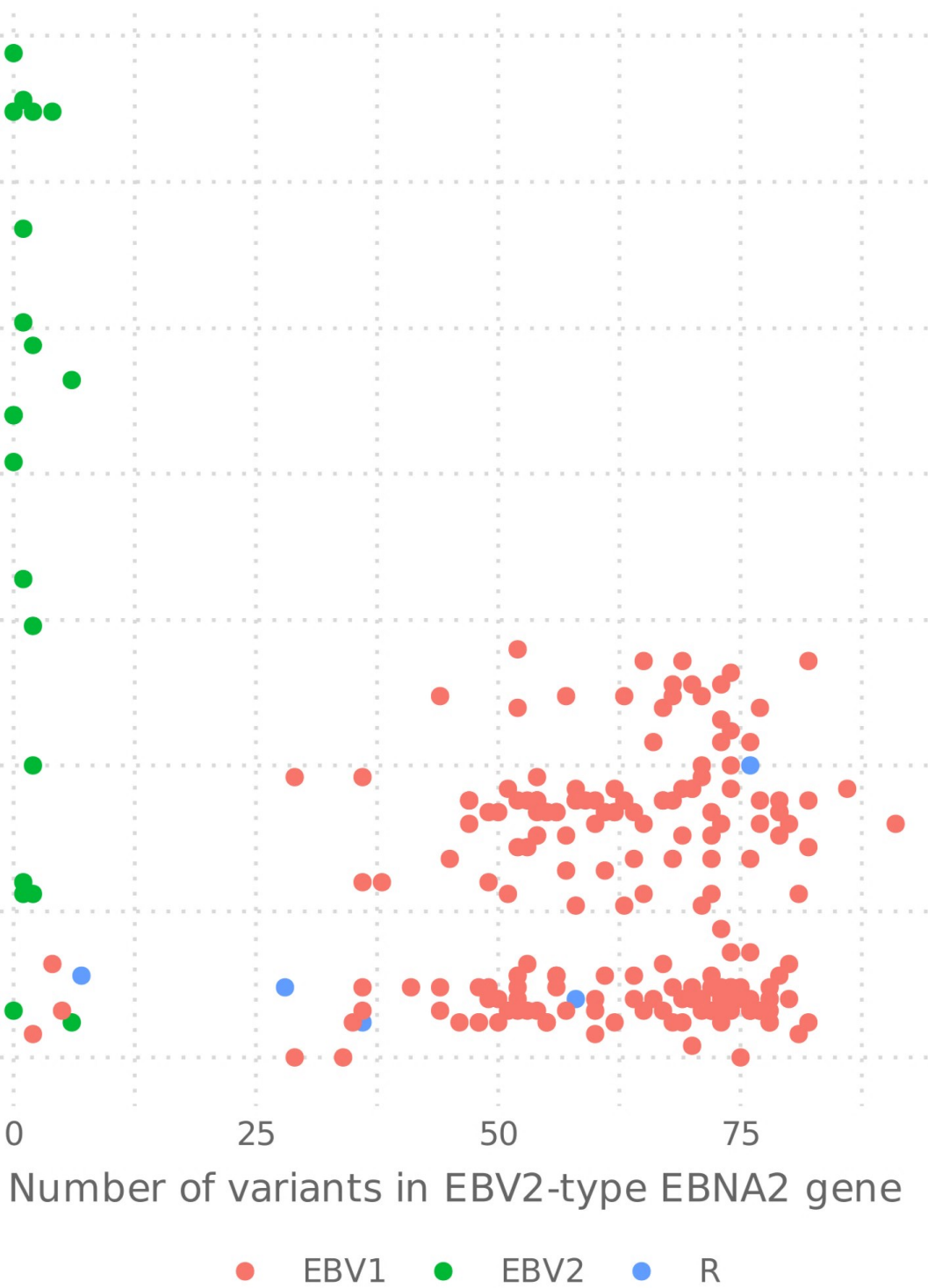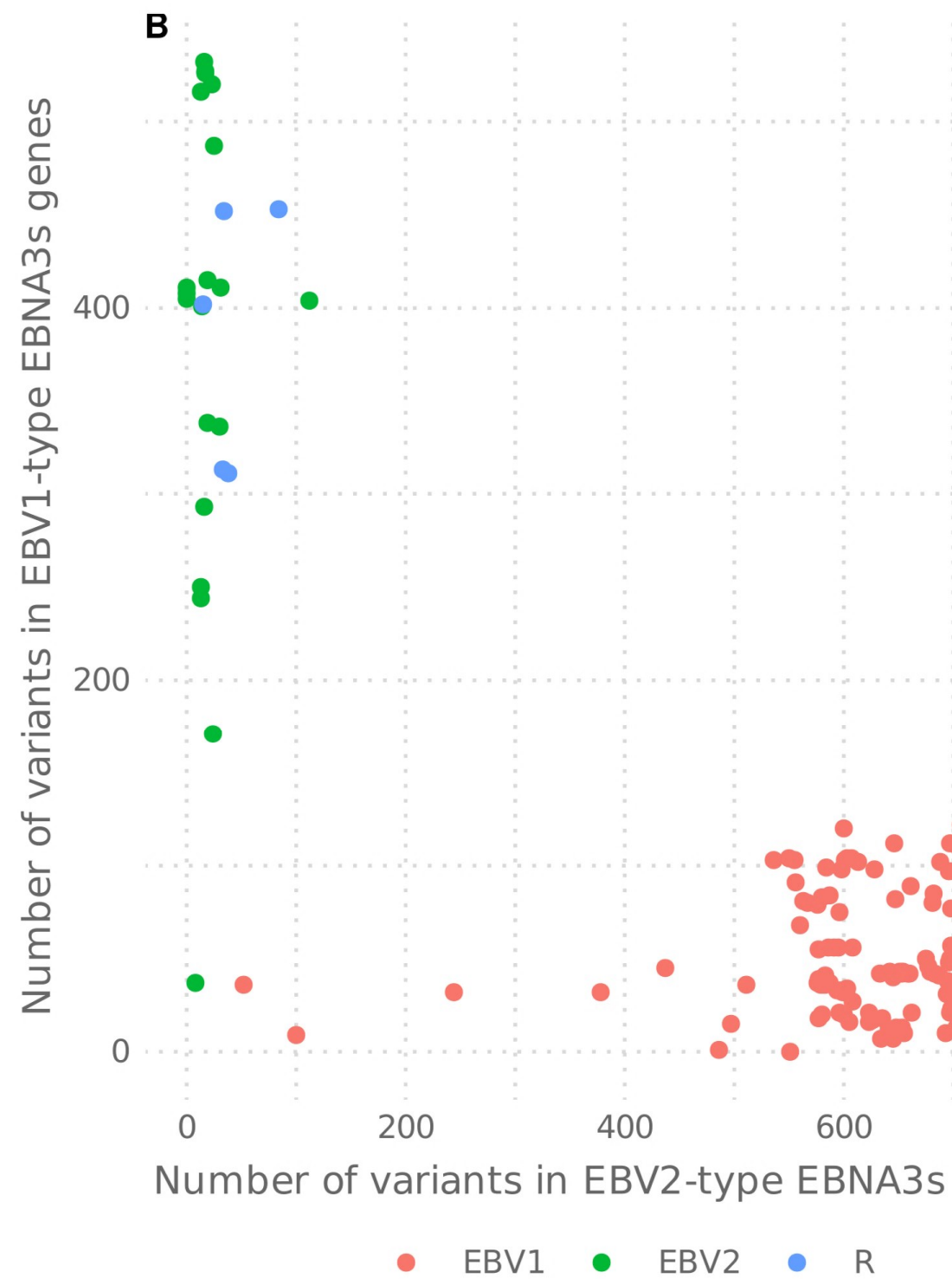

Supplement: Supplementary file 1 [file viruses-13-01172-s001.zip › Blazquez_SFig1.pdf]
